# Barriers associated with reduced physical activity in COPD patients*

**DOI:** 10.1590/S1806-37132014000500006

**Published:** 2014

**Authors:** Priscila Batista Amorim, Rafael Stelmach, Celso Ricardo Fernandes Carvalho, Frederico Leon Arrabal Fernandes, Regina Maria Carvalho-Pinto, Alberto Cukier

**Affiliations:** Heart Institute, University of São Paulo School of Medicine Hospital das Clínicas, São Paulo, Brazil; Department of Pulmonology, Heart Institute, University of São Paulo School of Medicine Hospital das Clínicas , São Paulo, Brazil; Heart Institute, University of São Paulo School of Medicine Hospital das Clínicas, São Paulo, Brazil; Department of Pulmonology, Heart Institute, University of São Paulo School of Medicine Hospital das Clínicas , São Paulo, Brazil; Department of Pulmonology, Heart Institute, University of São Paulo School of Medicine Hospital das Clínicas , São Paulo, Brazil; Department of Pulmonology, Heart Institute, University of São Paulo School of Medicine Hospital das Clínicas, São Paulo, Brazil

**Keywords:** Pulmonary disease, chronic obstructive, Activities of daily living, Exercise tolerance

## Abstract

**OBJECTIVE:**

To evaluate the ability of COPD patients to perform activities of daily living (ADL); to identify barriers that prevent these individuals from performing ADL; and to correlate those barriers with dyspnea severity, six-minute walk test (6MWT), and an ADL limitation score.

**METHODS:**

In COPD patients and healthy, age-matched controls, the number of steps, the distance walked, and walking time were recorded with a triaxial accelerometer, for seven consecutive days. A questionnaire regarding perceived barriers and the London Chest Activity of Daily Living (LCADL) scale were used in order to identify the factors that prevent the performance of ADL. The severity of dyspnea was assessed with two scales, whereas submaximal exercise capacity was determined on the basis of the 6MWT.

**RESULTS:**

We evaluated 40 COPD patients and 40 controls. In comparison with the control values, the mean walk time was significantly shorter for COPD patients (68.5 ± 25.8 min/day vs. 105.2 ± 49.4 min/day; p < 0.001), as was the distance walked (3.9 ± 1.9 km/day vs. 6.4 ± 3.2 km/day; p < 0.001). The COPD patients also walked fewer steps/day. The most common self-reported barriers to performing ADL were lack of infrastructure, social influences, and lack of willpower. The 6MWT distance correlated with the results obtained with the accelerometer but not with the LCADL scale results.

**CONCLUSIONS:**

Patients with COPD are less active than are healthy adults of a comparable age. Physical inactivity and the barriers to performing ADL have immediate implications for clinical practice, calling for early intervention measures.

## Introduction

There are several reasons for physical inactivity among healthy and diseased individuals. Common factors include technological advances that have influenced daily life, including the means of urban transportation; lack of time; being overweight; changes in the weather; lack of social support; and lack of motivation.^(^ 1 ^)^ Physical activity is recognized to have a beneficial role in the prevention of chronic diseases, such as systemic arterial hypertension, coronary artery disease, diabetes, osteoporosis, anxiety, and depression.^(^ 2 ^)^

Physical inactivity in patients with COPD was demonstrated in a study in which physical activity was objectively assessed by means of an accelerometer.^(^ 3 ^)^ The authors of the study demonstrated that COPD patients spend less time walking or standing than do sedentary elderly volunteers. In a study of patients with varying degrees of COPD severity,^(^ 4 ^)^ it was shown that physical activity is reduced in those with milder disease in comparison with smokers without COPD. One group of authors^(^ 5 ^)^ compared COPD patients from Brazil and Austria and showed that 23% of the Brazilian patients and half of the Austrian patients did not reach an average of 30 min of walking per day, which is the minimum recommended physical activity level.^(^ 1 ^)^ Few studies have investigated physical activity levels in patients with COPD in Brazil. 

Knowledge of the determinants and outcomes of physical activity in COPD patients allows the development of interventions designed to guide future research and improve the management of COPD. In a systematic review of the determinants of physical activity in patients with COPD,^(^ 6 ^)^ the authors found studies of clinical, functional, sociodemographic, and lifestyle factors. In addition, they criticized the quality of the evidence found in cross-sectional uncontrolled studies, stating that it did not allow them to establish causal associations and that it led to inconsistent results, the exception being that physical activity reduces exacerbations and mortality in COPD patients.^(^ 6 ^)^

Comparisons between different populations indicate that the factors that prevent individuals from performing activities of daily living (ADL) are heterogeneous. In a study of elderly individuals from a South American country, adherence to a physical activity intervention was 42.6%. Morbidity, poverty, and urban violence reduced adherence to the intervention, whereas retirement, a history of physical activity, and the presence of green areas in the neighborhood increased it.^(^ 7 ^)^ In a population-based study of individuals in the 40-79 year age bracket, self-reported inability to perform ADL was twice to four times as high in COPD patients as it was in individuals without the disease. Advanced age, poor health status, and anxiety/depression were associated with a higher probability of disability.^(^ 8 ^)^ In a sample of 9,415 adults in the USA, 9.6% of whom reported having COPD, 44.3% (vs. 27.5% of those who reported not having COPD) had difficulty in performing at least one ADL (60% having difficulty in performing practical/instrumental ADL), being less likely to be engaged in social activities and more likely to die.^(^ 9 ^)^ The aforementioned data suggest that it is necessary to identify the barriers to performing ADL in each population in order to increase the chance of success of ADL programs. 

In a systematic review aimed at identifying barriers to and facilitators of physical activity (including pulmonary rehabilitation) in patients with COPD, only a small number of studies met the inclusion criteria. Most (70%) of the studies included in the review were qualitative studies and had methodological problems, such as small sample size and poor description of data collection and analysis.^(^ 10 ^)^ Barriers identified included changing health status, personal problems, lack of support, external factors, smoking, and program-specific barriers. In one of the studies included in the aforementioned systematic review,^(^ 10 ^,^ 11 ^)^ the authors identified a plethora of barriers to participation in rehabilitation programs following hospitalization for COPD exacerbation. However, given that most of the barriers identified were subjective and were derived from interviews, it is difficult to use those barriers in the comparison of different groups. In fact, little is known about the barriers that prevent COPD patients from being more physically active or how to measure such barriers.^(^ 12 ^)^

Patients with COPD benefit from appropriate physical activity. Some of the benefits include increased exercise capacity, improvement in dyspnea, improved psychological and emotional status, improved quality of life, fewer emergency room visits,^(^ 2 ^)^ and reduced risk of exacerbation. The 2013 consensus statement on pulmonary rehabilitation established that patients with COPD should seek to increase their physical activity levels.^(^ 12 ^)^ Desired outcomes include reduced dyspnea, increased distance walked, and increased walk time, given that dyspnea, the distance walked, and the time spent walking are directly related to the level of physical activity. Accelerometers and pedometers can be used in order to measure and increase physical activity.^(^ 13 ^)^

In the present study, we hypothesized that daily physical activity levels are lower in patients with COPD than in healthy, age-matched controls, and that this is not exclusively due to symptoms or functional limitations, being also due to psychological, social, and cultural barriers. Our objective was to evaluate the ability of COPD patients to perform ADL, as well as to identify barriers that prevent such patients from being more physically active, in order to develop an effective program to encourage physical activity. 

## Methods

Patients with COPD^(^ 14 ^)^ were recruited from among those treated at the pulmonology outpatient clinic of our hospital. In parallel, healthy elderly individuals were selected for the control group, which comprised spouses of the patients with COPD and individuals treated at the geriatric outpatient clinic of our hospital. The inclusion criteria for COPD patients were as follows: age ≥ 50 years; FEV_1_ ≤ 60% of the predicted value before bronchodilator use; FEV_1_/FVC < 0.70; and stable medication use in the past 30 days. 

The exclusion criteria were as follows: clinical exacerbation in the past 30 days; long-term home oxygen therapy; musculoskeletal, cognitive, and mental disorders preventing patients from completing questionnaires, undergoing tests, or both; and limited mobility or other major comorbidity. The control group comprised gender- and age-matched volunteers, all of whom reported no lung disease and had normal pulmonary function test results. 

The present study was approved by the local research ethics committee, and all of the individuals who agreed to participate gave written informed consent. 

The first visit included the following:

1. In order to determine the severity of baseline dyspnea, we used the modified Medical Research Council (mMRC) scale, the score for which ranges from 0 to 4 (a higher score translating to a higher degree of dyspnea),^(^ 15 ^)^ and the Baseline Dyspnea Index (BDI),^(^ 16 ^)^ which includes three domains: functional impairment; magnitude of task; and magnitude of effort. Scores for each domain range from 0 to 4, the total score ranging from 0 (maximum dyspnea) to 12 (no dyspnea) ^(^ 16 ^)^
2. For objective measurement of physical activity, the participants were instructed to carry a triaxial accelerometer (PowerWalker; Yamax, Tokyo, Japan) in a trouser or shirt pocket.^(^ 17 ^)^ The device records the number of steps, the distance walked (in kilometers) and the time spent walking. The participants were instructed to use it daily for 7 consecutive days, removing it before taking a shower and before going to bed at night
3. We administered a questionnaire assessing perceived barriers to performing ADL: lack of time; social influences; lack of energy; lack of willpower; fear of injury; lack of ability; and lack of infrastructure.^(^ 18 ^)^ Three specific questions addressing each of the aforementioned domains are answered, the score for each answer ranging from 0 to 3. The maximum possible score for each domain is 9 points, and a score greater than or equal to 5 indicates a significant barrier
4. We also administered the Brazilian Portuguese version of the London Chest Activity of Daily Living (LCADL) scale, which assesses dyspnea during ADL in patients with COPD. The LCADL scale consists of 15 questions divided into four domains: self-care activities; domestic activities; physical activities; and leisure activities. The total score can range from 0 to 75 points, a higher score translating to greater limitations in ADL.^(^ 19 ^)^ The minimal detectable change for the LCADL scale score to measure the effect of interventions is of less than 3.88 points ^(^ 20 ^)^
5. In order to assess submaximal exercise capacity, we used the six-minute walk test (6MWT). The 6MWT is a submaximal test that determines the functional capacity of patients with chronic lung disease, being easy to perform, well tolerated, reproducible, and inexpensive.^(^ 21 ^)^ The first visit also included SpO_2_ measurement

In their second visit to the pulmonology outpatient clinic (which occurred 7 days after the first), the participants returned the accelerometer and underwent spirometry for pulmonary function testing, performed in accordance with international guidelines.^(^ 22 ^)^ All of the spirometric values analyzed in the present study were obtained without the use of a bronchodilator. 

### Statistical analysis

In order to calculate the sample size, we conducted a pilot study involving 5 patients with COPD and 5 controls. We found a 45% difference in the number of steps during three days of monitoring with the accelerometer, the standard deviation being 40%. Considering a power of 0.9 and a type I error of 0.05, we calculated that each group required at least 30 individuals. 

We performed a descriptive analysis of the groups. Variables with normal distribution were expressed as mean and standard deviation, whereas those with non-normal distribution were expressed as median, 95% CI, and interquartile range. The self-reported barriers to performing ADL were compared on the basis of the percentage of affirmative answers. The baseline characteristics were compared by the t-test or the chi-square test. Linear correlation analysis was performed with Pearson's and Spearman's correlation coefficients. The level of significance was set at 5%. The SigmaStat statistical package, version 3.5 (Systat Software Inc., San Jose, CA, USA), was used. 

## Results

A total of 92 individuals (48 COPD patients and 44 controls) were invited to participate in the present study. Of the 48 patients with COPD, 6 declined to participate, 1 had overlapping asthma and COPD, and 1 had borderline SpO_2_, requiring long-term home oxygen therapy. Of the 44 controls, 2 declined to participate and 1 reported to be receiving treatment for prostate cancer. 

A total of 81 individuals were included in the study, and 80 (40 COPD patients and 40 controls) completed it. One of the controls was excluded from the study because of lung function changes. The sociodemographic and functional characteristics of the COPD patients and controls are presented in Table 1. As expected, there was a significant difference between the two groups in terms of the proportion of smokers. Likewise, all pulmonary function parameters were found to be significantly decreased in the COPD group (Table 1). The median mMRC scale score in the COPD group was 2.0 (interquartile range, 1.0-3.0). On the basis of the spirometry results, 2.8%, 27.8%, 55.5%, and 13.9% of the COPD patients were classified as having mild COPD, moderate COPD, severe COPD, and very severe COPD, respectively.^(^ 20 ^)^ With regard to exercise capacity, the 6MWD was approximately 82 m shorter in the COPD group than in the control group, the difference being statistically significant (p < 0.001). The same was true for pre-6MWT SpO_2_ (94.3% in the COPD group vs. 97.2% in the control group; p < 0.001). 

**Table 1:** Demographic and functional characteristics of the COPD patients and controls, as well as their habits and six-minute walk test results.a

Variable	Groups	
	COPD	Control
	(n = 40)	(n = 40)
Gender		
Female	18 (45.0)	21 (52.5)
Male	22 (55.0)	19 (47.5)
Age, years	64.4 ± 7.7	66.7 ± 9.9
Smoking	39 (97.4)*	9 (22.5)
BMI, kg/m^2^	25.7 ± 3.5	25.8 ± 3.7
FVC, L^b^	2.5 ± 0.5*	3.4 ± 0.7
FVC, % predicted^b^	84.8 ± 17.9*	111.2 ± 14.5
FEV_1_, L^b^	1.1 ± 0.4*	2.5 ± 0.5
FEV_1_, % predicted^b^	47.1 ± 15.4*	109.1 ± 13.6
FEV_1_/FVC^b^	0.5 ± 0.1*	0.7 ± 0.1
6MWD, m	483.7 ± 70.8*	565.0 ± 78.8
Pre-6MWT SpO_2_, %	94.3*	97.2

BMI: body mass index; 6MWD: six-minute walk distance; and 6MWT: six-minute walk test. aValues expressed as n (%) or as mean ± SD. bValues obtained without the use of a bronchodilator. *p < 0.001

The level of physical activity was found to be significantly lower in the COPD group than in the control group. The number of steps per day was 6,251.0 ± 2,422.8 vs. 9,854.1 ± 4,736.6 in the control group (p < 0.001). In comparison with the control values, the mean walk time was significantly shorter for COPD patients (68.5 ± 25.8 min/day vs. 105.2 ± 49.4 min/day; p < 0.001), as was the distance walked (3.9 ± 1.9 km/day vs. 6.4 ± 3.2 km/day; p < 0.001). 

**Figure 1:**
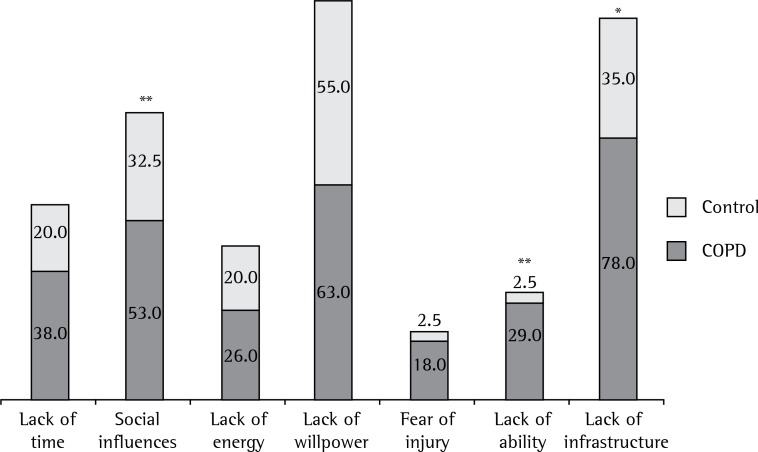
Questionnaire regarding perceived barriers to physical activity: proportion of participants with scores = 5 on each domain in the COPD and control groups.

Among the COPD patients, lack of infrastructure was the most common self-reported barrier to physical activity. Table 2 shows the scores obtained by the COPD patients and controls on the questionnaire regarding barriers to ADL. There were no significant differences between the COPD and control groups in terms of measures of central tendency. However, when divided by questionnaire values of intrinsic significance (5 points), approximately 80% of the patients with COPD and 35% of the controls (p < 0.001) reported that they did not perform physical activities because they had no access to exercise facilities or because they had no resources to exercise. Lack of willpower was the second most common self-reported barrier to performing ADL, reported by 63% of the patients with COPD and 55% of the controls, followed by social influences, reported by 53% of the patients with COPD and 32.5% of the controls (p < 0.05). The social influence domain includes having no one (e.g., family members or friends) to exercise with (or receiving no encouragement from family members or friends to exercise), as well as feeling embarrassed when performing physical activities. The remaining scores on the questionnaire regarding barriers to ADL are shown in Figure 1. A significant difference was found between the COPD and control groups regarding the lack of ability domain, indicating that patients with COPD feel that they are unable to perform ADL. 

**Table 2:** Scores on the questionnaire regarding perceived barriers to physical activity in the COPD and control groups.

Group	Domains						
	Lack of time	Social influences	Lack of energy	Lack of willpower	Fear of injury	Lack of ability	Lack of infrastructure
COPD							
Mean ± SD	3.2 ± 3.2	4.7 ± 1.7	3.1 ± 2.7	5.2± 1.7*	2.3 ± 2.5	3.0 ± 2.7	6.7 ± 2.5*
Median (IR)	2 (0/6)	5 (3/6)	3 (1/5)	5 (4/6)	2 (0/4)	3 (0/5)	8 (5/9)
Max/min	9/0	9/1	9/0	9/2	8/0	9/0	9/0
Control							
Mean ± SD	2.5 ± 2.9	3.4 ± 2.3	2.4 ± 2.5	4.1 ± 2.9	1.1 ± 1.4	1.7 ± 2.2	3.9 ± 3.0
Median (IR)	2 (0/4)	3 (2/5)	2 (0/4)	5 (1/6)	0 (0/2)	1 (0/3)	3 (2/6)
Max/min	9/0	9/0	9/0	9/0	6/0	9/0	9/0

IR: interquartile range; and Max/min: maximum/minimum. *p < 0.05

There were significant differences between the patients with COPD and the controls regarding their scores on all LCADL scale domains except domestic activities, with a significant impact on the total score (Table 3). These results are consistent with those obtained with the accelerometer, which showed that the patients with COPD performed less physical activity than did the controls. 

**Table 3:** London Chest Activity of Daily Living scale scores in the COPD and control groups.a

Variable	Group	
	COPD	Control
Self-care activities	6.2 ± 2.3*	4.1 ± 0.2
Domestic activities	6.8 ± 5.3	5.5 ± 1.5
Physical activities	4.1 ± 1.3*	2.3 ± 0.6
Leisure activities	4.4 ± 1.6*	3.1 ± 0.3
Score, points	21.5 ± 8.3*	14.9 ± 2.2
Score, %	32.9 ±11*	20.8 ± 2.0

aValues expressed as mean ± SD. *p < 0.001

**Figure 2:**
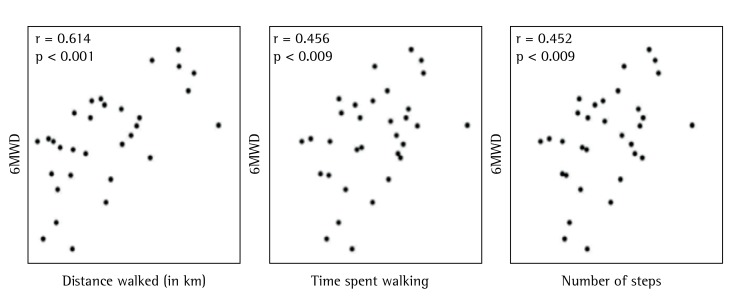
Correlation of the six-minute walk distance (6MWD) with the distance walked, the time spent walking, and the number of steps as measured by the accelerometer.

The 6MWD was significantly correlated with the time spent walking, the distance walked, and the number of steps as measured by the accelerometer (Figure 2). There was a trend toward a significant negative correlation between the 6MWD and the total LCADL scale score (R = −0.30; p = 0.08). In contrast, there was a trend toward a significant positive correlation between the BDI and the distance walked as measured by the accelerometer (R = 0.31; p = 0.06). There were no correlations of LCADL scale scores, FEV_1_, SpO_2_, and mMRC scale scores with the results obtained with the accelerometer. 

## Discussion

The results of the present study show that the level of physical activity as measured by an accelerometer is lower in patients with COPD than in healthy controls, as well as showing that the distance walked, the number of steps, and the walk time recorded daily by the accelerometer showed a significant linear correlation with the 6MWD. This difference between the two groups was identified by the LCADL scale as well. The results of the present study also showed that lack of infrastructure, lack of willpower, and social influences were the most common barriers that prevent COPD patients from being more physically active. 

With regard to ADL, our results corroborate those of previous studies.^(^ 23 ^)^ A literature review of COPD showed a significant reduction in the duration and intensity of ADL in patients with COPD when compared with healthy controls. The physical activity level of the controls in the present study was found to be similar to those of elderly individuals and smokers in other studies.^(^ 13 ^,^ 24 ^)^ In the aforementioned studies of COPD patients, most of the individuals were male. In the present study, the 80 participants were well distributed by gender and age, and most of the controls were spouses of the COPD patients, meaning that they were in the same social circle. Our findings reinforce the efficiency of accelerometers in determining the physical activity levels of COPD patients in a simple and easily repeatable manner. 

In the present study, the 6MWT confirmed the findings of other studies, showing in a sensitive manner that patients with COPD have lower exercise capacity, as well as confirming the correlation of the 6MWD (in m) with the performance of ADL as measured by the accelerometer over a greater number of days, better with the distance walked in kilometers in the present study, previously demonstrated with the use of a multiaxial accelerometer.^(^ 25 ^)^ Although the 6MWT is known to reflect the performance of ADL, it is more widely used in order to measure interventions, especially in research. In contrast, accelerometers, which are currently widely used, can be used in routine care. It should be emphasized that a low level of physical activity leads to a higher risk of mortality and hospitalization.^(^ 26 ^)^

The BDI tended to correlate with the distance walked as measured by the accelerometer. This suggests that the BDI is more sensitive in the evaluation of this relationship. However, daily dyspnea (as assessed by the LCADL scale) apparently did not affect the performance of ADL; no correlations were found between LCADL scale scores and the results obtained with the accelerometer. Nevertheless, although the LCADL is apparently better for self-assessed changes in COPD patients undergoing training programs than is the mMRC scale,^(^ 27 ^)^ the difference between the COPD patients and the controls in the present study was four times greater than the change obtained by such programs. 

Studies evaluating the reliability and sensitivity of the LCADL scale showed a weak but significant correlation between LCADL scores and shuttle walk test results.^(^ 28 ^)^ In the present study, the 6MWD tended to correlate with the total LCADL scale score, such a correlation having been demonstrated in a study in which the Brazilian Portuguese version of the scale was validated.^(^ 29 ^)^ A larger sample might be required in order to confirm these findings. 

One current challenge is to determine the external factors that influence the lack of physical activity. The most common self-reported barriers to physical activity in the general population are as follows^(^ 30 ^)^: not having enough time to exercise; not finding exercise enjoyable (finding it inconvenient to exercise); lack of self-motivation; finding exercise unpleasant; finding exercise boring; lack of confidence in the ability to be physically active (low self-efficacy); fear of being injured or having recently been injured; lack of self-management skills, such as the ability to set personal goals, monitor progress, or reward progress toward such goals; lack of encouragement, support, or companionship from family and friends; and lack of parks, sidewalks, bicycle trails, or safe and pleasant walking paths convenient to homes or offices. The US Centers for Disease Control and Prevention have sought to measure and encourage physical activity for 20 years. The questionnaire used in the present study in order to assess barriers to physical activity originated from US Centers for Disease Control and Prevention recommendations.^(^ 30 ^)^

Lack of infrastructure, social influences, and lack of ability were the most common self-reported barriers to physical activity in the COPD patients in the present study. However, it is of note that a large proportion of individuals in the control group also reported lack of infrastructure, lack of willpower, and social influences as barriers to physical activity. The lack of ability to perform physical activities might be directly related to insecurity. A study of 28 patients (22 males and 6 females) with COPD sought to identify, through interviews, the main barriers to and facilitators of physical activity after hospitalization.^(^ 11 ^)^ After systematization, the barriers were divided into three broad categories: health-related barriers; environment-related barriers; and self-related barriers. Health-related barriers included comorbidities, COPD (or COPD severity), and physical health or status. Environment-related barriers included the weather, (house) dust, and pollen, as well as transport difficulties (which were considered a major barrier to pulmonary rehabilitation) and financial difficulties, which were also related to the cost of transportation, especially for patients receiving home oxygen therapy (the cost of oxygen having also been reported). Self-related barriers included advanced age, lack of access to oxygen therapy, and problems related to physical activity/pulmonary rehabilitation programs. The authors reported the need for actively recognizing and overcoming barriers to physical activity and pulmonary rehabilitation.^(^ 11 ^)^

In addition to the severity of COPD, the small number of pulmonary rehabilitation centers in Brazil represents a real barrier; most of the COPD patients in the present study reported that they would engage in physical activity if they had more resources and more encouragement. However, the mere existence of pulmonary rehabilitation centers does not guarantee higher levels of physical activity. Not being able to perform ADL with someone who does not have COPD-because of the functional differences, this inability leading to a feeling of physical disability-can aggravate depression, which is common in patients with COPD. The fear and lack of knowledge of family members and friends regarding disability in patients with COPD also have a direct influence on perceived barriers to physical activity. 

The present study has limitations because it was a cross-sectional study with a small sample size. However, we used an objective instrument in order to assess the barriers to physical activity, and this aids in planning an intervention to change the sedentary lifestyle of COPD patients (and the consequences thereof). Physical inactivity and the barriers to physical activity in COPD patients have immediate implications for clinical practice, calling for early intervention measures. Physical exercise (e.g., walking) is recommended as a form of treatment for patients with COPD, allowing them to maintain a level of independence in ADL and making them more physically active, as well as reducing the impact of COPD and changing the prognosis of the disease. 

We conclude that patients with COPD are less active than healthy adults, and that lack of infrastructure, lack of willpower, and social influences are the main barriers to physical activity in patients with COPD. The time spent walking, the distance walked (in km), and the number of steps taken are objective, easily obtained measurements of physical inactivity in patients with COPD, being directly correlated with the 6MWD. Further studies, involving a larger number of patients and the use of more detailed questionnaires assessing limitations in and perceived barriers to ADL, are required for the planning of physical activity programs for patients with COPD.
