# Supplementary material for: Barriers associated with reduced physical activity in COPD patients
Source: J Bras Pneumol. 2014 Sep-Oct;40(5):504–12. doi: 10.1590/S1806-37132014000500006 (PMC4263331; doi:10.1590/S1806-37132014000500006)
Supplement: Translation

# Translation: Barreiras associadas à menor atividade física em portadores de DPOC*

**Authors:** Priscila Batista Amorim, Rafael Stelmach, Celso Ricardo Fernandes Carvalho, Frederico Leon Arrabal Fernandes, Regina Maria Carvalho-Pinto, Alberto Cukier

**Affiliations:** Instituto do Coração, Hospital das Clínicas, Faculdade de Medicina, Universidade de São Paulo, São Paulo (SP) Brasil; Divisão de Pneumologia, Instituto do Coração, Hospital das Clínicas, Faculdade de Medicina, Universidade de São Paulo, São Paulo (SP) Brasil; Hospital das Clínicas, Faculdade de Medicina, Universidade de São Paulo, São Paulo (SP) Brasil; Divisão de Pneumologia, Instituto do Coração, Hospital das Clínicas, Faculdade de Medicina, Universidade de São Paulo, São Paulo (SP) Brasil; Divisão de Pneumologia, Instituto do Coração, Hospital das Clínicas, Faculdade de Medicina, Universidade de São Paulo, São Paulo (SP) Brasil; Divisão de Pneumologia, Instituto do Coração, Hospital das Clínicas, Faculdade de Medicina, Universidade de São Paulo, São Paulo (SP) Brasil

**Keywords:** Doença pulmonar obstrutiva crônica, Atividades cotidianas, Tolerância ao exercício

## Abstract

**OBJETIVO:**

Avaliar a capacidade de portadores de DPOC em realizar atividades de vida diária (AVD), identificar barreiras que impedem a sua realização, e correlacionar essas barreiras com gravidade da dispneia, teste de caminhada de seis minutos (TC6) e um escore de limitação de AVD.

**MÉTODOS:**

Nos pacientes com DPOC e controles saudáveis pareados por idade, o número de passos, a distância percorrida e o tempo de caminhada foram registrados por um acelerômetro tridimensional durante sete dias consecutivos. Um questionário de barreiras percebidas e a escala *London Chest Activity of Daily Living* (LCADL) foram utilizados para identificar os fatores que impedem a realização de AVD. A dispneia foi medida por duas escalas distintas, e a capacidade física submáxima foi determinada com base no TC6.

**RESULTADOS:**

Foram avaliados 40 sujeitos com DPOC e 40 controles. Os pacientes com DPOC, comparados aos controles, realizaram menor tempo de caminhada (68,5 ± 25,8 min/dia vs. 105,2 ± 49,4 min/dia; p < 0,001), menor distância caminhada (3,9 ± 1,9 km/dia vs. 6,4 ± 3,2 km/dia; p < 0,001) e menor número de passos/dia. As principais barreiras referidas para realização de AVD foram falta de estrutura, influência social e falta de vontade. A distância caminhada no TC6 correlacionou-se com os resultados do acelerômetro, mas não os resultados da LCADL.

**CONCLUSÕES:**

Portadores de DPOC são menos ativos quando comparados a adultos saudáveis com idade comparável. O sedentarismo e as barreiras para a realização de AVD têm implicações imediatas na prática clínica, exigindo medidas precoces de intervenção.

## Introdução

Existem vários motivos para a população normal e a de pacientes se manterem sedentários. Fatores comuns incluem os avanços tecnológicos que têm influenciado o cotidiano, incluindo a forma de transporte urbano; a falta de tempo; o sobrepeso; as variações climáticas; a falta de suporte social; e a falta de motivação.^(^ 1 ^)^ O benefício da atividade física é reconhecido na prevenção de moléstias crônicas, como hipertensão arterial sistêmica, insuficiência coronariana, diabete, osteoporose, ansiedade e depressão.^(^ 2 ^)^

O sedentarismo de pacientes com DPOC ficou demonstrado por um estudo pelo qual a atividade física foi avaliada objetivamente pelo uso de um acelerômetro.^(^ 3 ^)^ Os autores demonstraram que os pacientes despendem menos tempo andando ou em pé, quando comparados a voluntários idosos sedentários. Em pacientes de diferentes graus de gravidade de DPOC,^(^ 4 ^)^ mostrou-se que, nos estágios mais leves da doença, esses reduzem sua atividade em relação a fumantes sem DPOC. Um grupo de autores^(^ 5 ^)^ demonstrou ainda que 23% dos brasileiros contra a metade dos austríacos não atingiram o limite de 30 minutos de caminhada por dia, índice mínimo de nível de atividade recomendado.^(^ 1 ^)^ O nível de atividade física do portador de DPOC brasileiro ainda é pouco estudado.

O conhecimento sobre os determinantes e os resultados de um programa de atividade física em pacientes com DPOC permite projetar intervenções para orientar futuras pesquisas e melhorar o manejo desses pacientes. Em uma revisão sistemática sobre esses determinantes nessa população,^(^ 6 ^)^ foram encontrados estudos sobre fatores clínicos, funcionais e sociodemográficos, assim como em relação ao estilo de vida. A revisão critica a qualidade das evidências encontradas nos estudos transversais não controlados que não permitiram uma associação causal ou que levaram a resultados inconsistentes, exceto em relação à diminuição de exacerbações e da mortalidade.^(^ 6 ^)^

Comparações entre diferentes populações indicam que os fatores limitantes a atividades de vida diária (AVD) não são homogêneos. Em um estudo realizado com uma população sul-americana idosa, houve a adesão de 42,6% a uma intervenção que incentivava as AVD. Os fatores que influenciaram a adesão foram variados: morbidades, pobreza e violência urbana diminuíam a adesão, enquanto aposentadoria, história de prática de atividade física e existência de áreas verdes na vizinhança a aumentavam. ^(^ 7 ^)^ Portadores de DPOC relataram um grau de incapacidade para a realização de AVD de duas a quatro vezes maior do que não portadores em estudo populacional de pacientes com idade entre 40 e 79 anos. Ter idade avançada, ter condições precárias de saúde e sofrer de ansiedade e/ou depressão foram fatores relacionados a maior probabilidade de incapacidade.^(^ 8 ^)^ Numa amostra de 9.415 adultos nos EUA, dos quais 9,6% diziam ser portadores de DPOC, 44,3% (vs. 27,5% daqueles sem autorrelato de DPOC) tinham pelos menos uma dificuldade para a realização de AVD, principalmente aquelas de ordem prática/instrumental (60% as referiam), levando a um menor engajamento nas atividades sociais e uma maior probabilidade de morte.^(9) ^Esses dados sugerem que é necessário identificar essas barreiras em cada população para aumentar a chance de sucesso de programas de incentivo à realização de AVD.

Em uma revisão sistemática realizada com o propósito de avaliar barreiras e fatores facilitadores para a realização de atividades física em pacientes com DPOC, incluindo a reabilitação pulmonar, encontrou-se um número pequeno de estudos. A maioria deles era qualitativo (70%) e com problemas metodológicos, como amostra pequena e descrição inadequada dos dados e/ou de sua análise.^(^ 10 ^)^ As principais barreiras identificadas foram a mudança do status de saúde, problemas pessoais, falta de apoio, fatores externos, tabagismo e barreiras dos próprios programas. Em um dos estudos incluídos na revisão sistemática,^(^ 10 ^,^ 11 ^)^ a dificuldade de participação em programas de reabilitação após a internação por exacerbação de DPOC foi justificada pela pletora de barreiras encontradas. Entretanto, como a maior parte das barreiras identificadas é subjetiva, derivada de entrevistas, é difícil utilizá-las comparando-se grupos diferentes. De fato, pouco se sabe sobre as barreiras que impedem a população de DPOC tornar-se mais ativa ou como mensurá-las.^(^ 12 ^)^

Os portadores de DPOC se beneficiam da manutenção de atividade física adequada. O aumento da capacidade de exercício, a melhora da dispneia, assim como dos aspectos psicoemocionais e da qualidade de vida, a menor procura a serviços de emergência^(^ 2 ^)^ e a redução dos riscos de exacerbação foram alguns dos benefícios referidos. O novo consenso de reabilitação pulmonar estabeleceu que o aumento do nível de atividade física é uma meta a ser perseguida em DPOC.^(^ 12 ^)^ Diminuir a dispneia e aumentar a distância e o tempo de caminhada, fatores diretamente relacionados ao menor nível de atividade, são desfechos desejados. Acelerômetros e pedômetros podem ser utilizados para facilitar o planejamento para o aumento da realização de AVD.^(^ 13 ^)^

No presente estudo, as hipóteses foram as seguintes: portadores de DPOC realizam menos atividades físicas no dia a dia do que controles com o mesmo perfil demográfico e que esse fato não apenas se associa a sintomas ou a limitação funcional, mas também a barreiras psicológicas, sociais e culturais. Nosso objetivo foi conhecer o nível de realização de AVD nessa população, as barreiras e limitações que impedem esses pacientes de serem mais ativos fisicamente para, futuramente, elaborar um programa efetivo de estímulo a atividade física.

## Métodos

Pacientes portadores de DPOC^(^ 14 ^)^ foram recrutados do ambulatório de pneumologia do nosso hospital. Paralelamente, idosos saudáveis foram selecionados para compor o grupo controle, sendo uma parcela desse grupo de cônjuges dos portadores de DPOC e outra proveniente do ambulatório de geriatria. Os critérios de inclusão para os portadores de DPOC foram ter idade ≥ 50 anos, apresentar VEF_1_ ≤ 60% do valor previsto antes do uso de broncodilatador, apresentar uma relação VEF_1_/CVF < 0,70 e estar em uso de medicação estável nos últimos 30 dias. 

Foram excluídos do protocolo pacientes que apresentaram exacerbação clínica nos últimos 30 dias, os dependentes de oxigenoterapia domiciliar prolongada, que apresentassem alterações musculoesqueléticas, cognitivas e mentais que impedissem o preenchimento de questionários e/ou realização dos testes, e os que apresentassem limitada mobilidade ou outra comorbidade maior. Os voluntários do grupo controle foram incluídos pareados por sexo e idade com o grupo de estudo. Todos negavam ser portadores de quaisquer doenças pulmonares e apresentavam resultados normais na prova de função pulmonar.

O estudo foi aprovado pela comissão de ética em pesquisa da instituição, e todos os participantes aceitaram participar da pesquisa e assinaram o termo de consentimento livre e esclarecido.

Durante a primeira visita, foram realizados os seguintes procedimentos:

a) Para avaliar a gravidade da dispneia basal, foram aplicados a escala modificada do *Medical Research Council* (mMRC), que varia de 0 a 4 pontos (quanto maior o valor, pior o grau de dispneia),^(^ 15 ^)^ e o *Baseline Dyspnea Index* (BDI),^(^ 16 ^)^ que inclui três domínios: dispneia para esforços fixos usuais, dispneia para atividades de trabalho e dispneia para esforços mais intensos. Esses domínios são avaliados por meio de notas que podem variar de 0 a 4 e cujo resultado final varia de nota 0 (dispneia máxima) a 12 (sem falta de ar).^(^ 16 ^)^
b) Para medir a realização de AVD objetivamente, os participantes da pesquisa foram instruídos a usar um acelerômetro (PowerWalker; Yamax, Tóquio, Japão) com sensor tridimensional no bolso da calça ou da camisa.^(^ 17 ^)^ Esse equipamento registra o número de passos, a distância em quilômetros e o tempo de caminhada. Os participantes foram orientados a utilizá-lo diariamente durante 7 dias consecutivos, retirando-o apenas na hora do banho e à noite ao dormir.
c) Foi aplicado o questionário de barreiras percebidas, que avalia sete barreiras que impedem o paciente de realizar atividades físicas: falta de tempo, influência social, falta de energia, falta de vontade, medo de lesão, falta de habilidade e falta de estrutura.^(18) ^Três perguntas especificas sobre cada um desses domínios são respondidas, sendo valoradas de 0 a 3 pontos cada uma. O escore final pode chegar a 9 pontos, e um resultado maior ou igual a 5 representa uma barreira significativa.
d) Também foi aplicada a versão brasileira *do London Chest Activity of Daily Living* (LCADL), que avalia a dispneia durante a realização de AVD em pacientes com DPOC. São 15 questões divididas em quatro domínios: cuidados pessoais, atividades domésticas, atividades físicas e lazer. O escore total pode variar de 0 até 75 pontos, sendo que quanto mais alto, maior é a limitação às AVD.^(^ 19 ^)^ O mínimo valor significante do LCADL para medir o efeito de intervenções é de menos 3,88 pontos.^(20)^
e) Para avaliar a capacidade física submáxima foi aplicado o teste de caminhada de seis minutos (TC6). Trata-se de um teste que determina a capacidade funcional de pneumopatas crônicos, sendo de fácil execução, bem tolerado, reprodutível e de baixo custo.^(^ 21 ^)^ Durante essa primeira visita, também foi verificada a SpO_2_.

Após 7 dias da visita inicial, os participantes retornavam ao ambulatório para devolver o acelerômetro e realizar a espirometria para a avaliação da função pulmonar, de acordo com as diretrizes internacionais.^(^ 22 ^)^ Os valores analisados de pacientes e controles foram obtidos sem o uso de broncodilatador.

### Análise estatística

Para o cálculo do número de pacientes necessários realizou-se um estudo piloto inicial com 5 portadores de DPOC e 5 controles. A diferença encontrada no número de passos durante a monitorização com o acelerômetro (3 dias) foi de 45%, com um desvio-padrão de 40%. Considerando-se um poder de 0,9 e um erro alfa de 0,05, cada grupo deveria ter no mínimo 30 participantes.

A análise descritiva dos grupos foi realizada e apresentada em média e desvio-padrão, no caso de variáveis com distribuição normal, ou mediana e IC95% e intervalo interquartílico nas de apresentação não normal. A avaliação de barreiras foi comparada pelo percentual de positividade referida. As características basais foram comparadas usando-se o teste t ou o teste do qui-quadrado. A análise de correlação linear foi feita usando os coeficientes de Pearson e de Spearman. O nível de significância adotado foi de 5%. Utilizou-se o programa SigmaStat, versão 3.5 (Systat Software Inc., San Jose, CA, EUA). 

## Resultados

Foram convidados para participar do estudo 92 indivíduos (sendo 48 portadores de DPOC e 44 do grupo controle). Desses, 6 portadores de DPOC desistiram de participar da pesquisa, 1 apresentava sobreposição (diagnóstico de asma e DPOC), e 1 apresentava SpO_2_ limítrofe, com indicação de uso de oxigenoterapia domiciliar prolongada. Do grupo controle, 2 indivíduos desistiram de participar da pesquisa, e 1 referiu câncer de próstata em tratamento.

Foram incluídos 81 participantes, e 80 concluíram a pesquisa (40 portadores de DPOC e 40 do grupo controle). Um participante foi excluído do grupo controle por apresentar alterações na função pulmonar. As características sociodemográficas e funcionais dos pacientes e controles estão apresentadas na Tabela 1. Como esperado, o percentual de tabagistas foi estatisticamente diferente entre os grupos. Do mesmo modo, todos os parâmetros de função pulmonar dos indivíduos com DPOC estavam diminuídos de maneira significante (Tabela 1). Na escala MMRC, a mediana (intervalo interquartílico) dos pacientes no grupo DPOC foi de 2,0 (1,0-3,0) pontos. No grupo DPOC, após a estratificação pela espirometria, o grau de DPOC foi considerado leve, em 2,8% dos pacientes; moderada, em 27,8%; grave, em 55,5%; e muito grave, em 13,9%.^(^ 20 ^)^ Quanto à capacidade de exercício, representada pelo TC6, houve igualmente diferença significante: os pacientes do grupo DPOC andaram cerca de 82 m a menos que os do grupo controle (p < 0,001). O mesmo foi observado com a SpO_2_ ao início do TC6 (grupo DPOC, 94,3% vs. grupo controle, 97,2%; p < 0,001).

**Tabela 1:** Características demográficas e funcionais, hábitos e resultados do teste de caminhada de seis minutos nos grupos DPOC e controle.a

Variáveis	Grupos	
	DPOC	Controle
	(n = 40)	(n = 40)
Sexo		
Feminino	18 (45,0)	21 (52,5)
Masculino	22 (55,0)	19 (47,5)
Idade, anos	64,4 ± 7,7	66,7 ± 9,9
Tabagismo	39 (97,4)*	9 (22,5)
IMC, kg/m^2^	25,7 ± 3,5	25,8 ± 3,7
CVF, L^b^	2,5 ± 0,5*	3,4 ± 0,7
CVF, % previsto^b^	84,8 ± 17,9*	111,2 ± 14,5
VEF_1_, L^b^	1,1 ± 0,4*	2,5 ± 0,5
VEF_1_, % previsto^b^	47,1 ± 15,4*	109,1 ± 13,6
VEF_1_/CVF^b^	0,5 ± 0,1*	0,7 ± 0,1
Distância TC6, m	483,7 ± 70,8*	565,0 ± 78,8
SpO_2_ inicial TC6, %	94,3*	97,2

IMC: índice de massa corpórea; e TC6: teste de caminhada de 6 minutos

a Valores expressos em n (%) ou em média ± dp

b Valores sem o uso de broncodilatador

* p < 0,001.

Os pacientes com DPOC apresentam um nível de realização de AVD significantemente menor quando comparados com os sujeitos do grupo controle. O número de passos por dia foi de 6.251,0 ± 2.422,8 vs. 9.854,1 ± 4.736,6 no grupo controle (p < 0,001). Na comparação dos grupos DPOC e controle, respectivamente, o dispêndio de tempo caminhando foi de 68,5 ± 25,8 min/dia e 105,2 ± 49,4 min/dia (p < 0,001). A distância percorrida também foi significativamente diferente entre os dois grupos (3,9 ± 1,9 km/dia vs. 6,4 ± 3,2 km/dia; p < 0,001).

A falta de estrutura mostrou ser a principal barreira para a realização de atividades físicas entre os pacientes com DPOC. Os valores do questionário de barreiras, por grupo, estão mostrados na Tabela 2. Não houve diferença estatisticamente significante entre os grupos nas medidas de tendência central. Entretanto, quando divididos por valores de significância intrínseca do questionário (5 pontos), cerca de 80% dos portadores de DPOC relataram que não ter realizado atividades físicas por não ter acesso a um local para praticar exercícios ou recursos contra 35% no grupo controle (p < 0,001). A falta de vontade veio em segundo lugar, com 63% e, em terceiro lugar, a influência social, com 53% no grupo DPOC vs. 55% e 32,5% no grupo controle, respectivamente (p < 0,05). A falta de influência social refere-se à falta de parceiros ou estímulo desses para se exercitar, bem como sentir-se envergonhado ao realizar atividades físicas. Os demais valores do questionário de barreiras estão mostrados na Figura 1. A falta de habilidade também foi significativamente diferente entre os grupos, indicando que portadores de DPOC sentem-se incompetentes para a realização de AVD.

**Tabela 2:** Pontuação do questionário de barreiras por grupo.

Grupos	Domínios						
	Falta de tempo	Influência social	Falta de energia	Falta de vontade	Medo de lesão	Falta de habilidade	Falta de estrutura
DPOC							
Média ± dp	3,2 ± 3,2	4,7 ± 1,7	3,1 ± 2,7	5,2 ± 1,7*	2,3 ± 2,5	3,0 ± 2,7	6,7 ± 2,5*
Mediana (II)	2 (0/6)	5 (3/6)	3 (1/5)	5 (4/6)	2 (0/4)	3 (0/5)	8 (5/9)
Máx/mín	9/0	9/1	9/0	9/2	8/0	9/0	9/0
Controle							
Média ± dp	2,5 ± 2,9	3,4 ± 2,3	2,4 ± 2,5	4,1 ± 2,9	1,1 ± 1,4	1,7 ± 2,2	3,9 ± 3,0
Mediana (II)	2 (0/4)	3 (2/5)	2 (0/4)	5 (1/6)	0 (0/2)	1 (0/3)	3 (2/6)
Máx/mín	9/0	9/0	9/0	9/0	6/0	9/0	9/0

II: intervalo interquartílico; e Max/mín: máximo/mínimo

* p < 0,05.

**Figura 1:** Questionário de barreiras: proporção de participantes com valores = 5 pontos por domínio nos grupos estudados.

Os resultados da escala de AVD mensurada pelo LCADL mostraram diferenças estatisticamente significantes entre os portadores de DPOC e os controles para quase todas as AVD, exceto para as domésticas, com impacto significativo no escore total (Tabela 3). Esses resultados corroboram a menor atividade física dos pacientes medida pelo acelerômetro em relação aos controles.

**Tabela 3:** Resultados do London Chest Activity of Daily Living por grupo.a

Variáveis	Grupos	
	DPOC	Controle
Cuidados pessoais	6,2 ± 2,3*	4,1 ± 0,2
Atividades domésticas	6,8 ± 5,3	5,5 ± 1,5
Atividade física	4,1 ± 1,3*	2,3 ± 0,6
Lazer	4,4 ± 1,6*	3,1 ± 0,3
Escore, pontos	21,5 ± 8,3*	14,9 ± 2,2
Escore, %	32,9 ±11*	20,8 ± 2,0

a Valores expressos em média ± dp

* p < 0,001.

O TC6 correlacionou-se de maneira estatisticamente significante com o tempo de caminhada, a distância e o número de passos mensurados pelo acelerômetro (Figura 2). A correlação do TC6 e o LCADL (total de pontos) mostrou uma tendência à significância negativa (R = −0,30, p = 0,08). O BDI e a distância medida pelo acelerômetro mostraram uma tendência de correlação positiva (R = 0,31; p = 0,06). Não houve correlações dos resultados de LCADL, VEF_1_, SpO_2_ e MMRC com os resultados obtidos com o acelerômetro.

**Figura 2:** Correlação da distância percorrida no teste de caminhada de seis minutos (DTC6) com a distância percorrida, tempo de caminhada e número de passos mensurados pelo acelerômetro.

## Discussão

No presente estudo, os resultados mostram que pacientes com DPOC têm um nível de AVD, medido por um sensor de movimento, inferior ao da população controle, e que a distância, o número de passos e o tempo de caminhada diários registrados pelo acelerômetro apresentaram uma correlação linear estatisticamente significante com o TC6. O questionário LCADL igualmente identificou essa diferença entre os grupos. Os resultados também mostraram que a falta de estrutura, a falta de vontade e a influência social foram as principais barreiras/motivos para essa população não ser mais ativa fisicamente.

No que se refere a AVD, esses resultados reforçam o que já foi apresentado em estudos prévios.^(^ 23 ^)^ Uma revisão bibliográfica de pacientes com DPOC mostrou uma significante redução da duração e da intensidade da realização de AVD quando esses foram comparados a controles saudáveis. O nível de atividade física dos controles no presente estudo também é semelhante ao de outros estudos com idosos e tabagistas. ^(^ 13 ^,^ 24 ^)^ Nos estudos com portadores de DPOC, a maioria dos indivíduos era do sexo masculino. No presente estudo, os 80 indivíduos estavam bem distribuídos tanto por sexo quanto por faixa etária, e a maioria dos controles era formada por conjugues dos pacientes com DPOC, ou seja, do mesmo convívio social. Nossos achados reforçam a eficiência dos acelerômetros na detecção do menor nível de AVD em portadores de DPOC de maneira simples e de fácil repetição.

O TC6 confirmou achados da literatura, mostrando de forma sensível que pacientes com DPOC têm uma menor capacidade ao exercício. Reiterou também a boa correlação da distância caminhada no TC6 (em m) com a realização de AVD registrada em sete dias. No presente estudo, a melhor correlação do TC6 foi com a distância caminhada em quilômetros medida com o acelerômetro e não como o tempo de caminhada, como demonstrado anteriormente.^(^ 25 ^)^ Apesar de o TC6 sabidamente refletir a realização de AVD, seu uso tem uma maior aplicação para medir intervenções, especialmente em pesquisa. Já os acelerômetros, atualmente bastante difundidos, podem ser utilizados na assistência cotidiana. Devemos lembrar que o baixo nível de realização de AVD leva a um maior risco de mortalidade e de hospitalização.^(^ 26 ^)^

O BDI mostrou uma tendência de correlação com a distância medida pelo acelerômetro, indicando que esse índice pode ser mais sensível que a escala mMRC na avaliação da relação dispneia e AVD. Entretanto, a dispneia diária durante a realização de AVD registradas pelo LCADL não pareceu interferir, já que não foram encontradas correlações entre o LCADL e os dados obtidos com o acelerômetro. Por outro lado, os valores de LCADL encontrados no presente estudo reforçam que esse é um instrumento melhor que a escala mMRC, tanto para a avaliação de pacientes submetidos a programas de treinamento,^(^ 27 ^)^ quanto na situação basal: os pacientes avaliados apresentavam resultados quatro vezes piores que os controles.

Em trabalhos prévios para avaliar a confiabilidade e sensibilidade do LCADL, houve uma correlação fraca porém estatisticamente significante com o *shuttle walk test*.^(^ 28 ^)^ No presente estudo, o TC6 mostrou uma tendência de correlação com o escore total do LCADL, já demonstrada na validação para o português desse instrumento.^(^ 29 ^)^ Talvez seja necessária uma casuística maior para que possamos confirmar esses achados.

Um desafio atual é conhecer quais são os fatores externos que podem influenciar a falta de prática de atividades física. As causas mais comuns citadas pela população em geral são as seguintes^(^ 30 ^)^: não ter tempo suficiente para realizar exercícios; não gostar de fazer exercícios (achar inconveniente); não ter automotivação; achar desagradável; achar chato; ter baixa autoeficácia, ou seja, ter falta de confiança na sua capacidade de ser fisicamente ativo; ter medo de se machucar ou por ter se machucado recentemente; não ter habilidade pessoal para definir objetivos, monitorar o progresso ou entender as recompensas obtidas ao alcançar os objetivos; não ter incentivo, apoio ou companheirismo por parte de familiares ou amigos; e não haver parques, calçadas, ciclovias, ou percursos pedestres convenientes, seguros e agradáveis próximos a suas casas ou locais de trabalho. Nos EUA, os *Centers for Disease Control and Prevention* procuram medir e incentivar a prática de atividades físicas há 20 anos. O questionário de barreiras utilizado no presente estudo originou-se dessas recomendações.^(^ 30 ^)^

A falta de estrutura, a influência social e a falta de habilidades foram as maiores barreiras para a prática de atividades físicas identificadas pelos portadores de DPOC no presente estudo. Entretanto, deve-se notar que uma grande proporção dos indivíduos no grupo controle referiu a falta de estrutura, a falta de vontade e a influência social como barreiras, de forma semelhante. A ausência de habilidade para a realização de uma atividade física pode estar diretamente relacionada à insegurança. Um estudo com 28 pacientes (22 homens e 6 mulheres) portadores de DOPC procurou identificar, através de entrevistas, as principais barreiras e facilitadores para a realização de atividades físicas após uma internação hospitalar.^(^ 11 ^)^ Após a sistematização, as barreiras foram divididas em três grandes categorias: saúde, meio ambiente e individual. Na categoria saúde, incluem-se as comorbidades, a própria DPOC (sua gravidade) e a saúde ou status físico. Na categoria meio ambiente, destacaram-se o clima, a presença de poeiras (domiciliar) ou polens, assim como dificuldades de transporte (considerada uma grande barreira para reabilitação pulmonar) e/ou dificuldades financeiras, também relacionadas ao custo do transporte, especialmente se o indivíduo é usuário de oxigênio domiciliar (o custo do oxigênio também foi referido). Na categoria individual, foram relatadas a idade avançada, a falta de acesso ou a prescrição de oxigenoterapia e problemas em seguir um programa de atividades físicas ou de reabilitação. Os autores sugerem ser necessário reconhecer ativamente as barreiras e tentar vencê-las para garantir o aumento da realização de AVD ou a participação na reabilitação pulmonar.^(^ 11 ^)^

Tanto quanto a gravidade da DPOC, o número reduzido de centros de reabilitação no país voltados para doentes pulmonares representa uma barreira real, já que a maioria dos pacientes do presente estudo relatou voluntariamente que realizariam atividades físicas se tivessem mais estrutura e mais incentivo. Entretanto, a simples existência desses centros não garante que se consiga mudar o nível de realização de AVD. Não poder compartilhar a AVD com uma pessoa não portadora de DPOC, dada as diferenças funcionais, levando a uma sensação de incompetência física, pode ser um fator agravador do quadro de depressão que frequentemente associa-se à DPOC. O medo e/ou a falta de conhecimento de familiares e amigos em relação à incapacidade do portador de DPOC também influenciam diretamente essas barreiras.

O presente estudo tem limitações por tratar-se de estudo transversal, com casuística pequena. Entretanto, utiliza um instrumento objetivo para medir as barreiras, o que ajuda o planejamento de como interferir no sedentarismo dos portadores de DPOC e suas consequências. Diagnosticar o nível de sedentarismo e as barreiras para a realização de atividades físicas tem implicações imediatas na prática clínica para que se criem medidas de intervenção precoces nesse grupo de pacientes. A busca pela independência através da manutenção das AVD indica a prática de exercícios físicos (caminhada, por exemplo) como tratamento, tornando esses pacientes mais ativos fisicamente, diminuindo o impacto da própria DPOC e modificando o prognóstico.

Concluímos que portadores de DPOC são menos ativos quando comparados a adultos saudáveis, e que a falta de estrutura, a falta de vontade e influência social são as principais barreiras para a prática de atividades físicas em pacientes com DPOC. O tempo de caminhada, a distância percorrida em km e o número de passos dados são medidas objetivas, registradas de maneira simples, do sedentarismo mórbido desses pacientes, com correlação direta com o TC6. Novos estudos com um número maior de pacientes, assim como questionários de barreiras e de limitações mais detalhados, são necessários para o planejamento da prática de atividades físicas em portadores de DPOC.
